# The Anti-Sigma Factor TcdC Modulates Hypervirulence in an Epidemic BI/NAP1/027 Clinical Isolate of *Clostridium difficile*

**DOI:** 10.1371/journal.ppat.1002317

**Published:** 2011-10-13

**Authors:** Glen P. Carter, Gillian R. Douce, Revathi Govind, Pauline M. Howarth, Kate E. Mackin, Janice Spencer, Anthony M. Buckley, Ana Antunes, Despina Kotsanas, Grant A. Jenkin, Bruno Dupuy, Julian I. Rood, Dena Lyras

**Affiliations:** 1 Department of Microbiology, Monash University, Clayton, Victoria, Australia; 2 Division of Infection and Immunity, FBLS Glasgow Biomedical Research Centre, University of Glasgow, Glasgow, United Kingdom; 3 Division of Biology, Kansas State University, Manhattan, Kansas, United States of America; 4 Laboratoire Pathogenèse des Bactéries Anaérobies, Institut Pasteur, Paris, France; 5 Department of Infectious Diseases, Southern Health, Monash Medical Centre, Clayton, Victoria, Australia; The University of Texas-Houston Medical School, United States of America

## Abstract

Nosocomial infections are increasingly being recognised as a major patient safety issue. The modern hospital environment and associated health care practices have provided a niche for the rapid evolution of microbial pathogens that are well adapted to surviving and proliferating in this setting, after which they can infect susceptible patients. This is clearly the case for bacterial pathogens such as Methicillin Resistant *Staphylococcus aureus* (MRSA) and Vancomycin Resistant *Enterococcus* (VRE) species, both of which have acquired resistance to antimicrobial agents as well as enhanced survival and virulence properties that present serious therapeutic dilemmas for treating physicians. It has recently become apparent that the spore-forming bacterium *Clostridium difficile* also falls within this category. Since 2000, there has been a striking increase in *C. difficile* nosocomial infections worldwide, predominantly due to the emergence of epidemic or hypervirulent isolates that appear to possess extended antibiotic resistance and virulence properties. Various hypotheses have been proposed for the emergence of these strains, and for their persistence and increased virulence, but supportive experimental data are lacking. Here we describe a genetic approach using isogenic strains to identify a factor linked to the development of hypervirulence in *C. difficile*. This study provides evidence that a naturally occurring mutation in a negative regulator of toxin production, the anti-sigma factor TcdC, is an important factor in the development of hypervirulence in epidemic *C. difficile* isolates, presumably because the mutation leads to significantly increased toxin production, a contentious hypothesis until now. These results have important implications for *C. difficile* pathogenesis and virulence since they suggest that strains carrying a similar mutation have the inherent potential to develop a hypervirulent phenotype.

## Author Summary

Hospital infections are increasingly being recognised as a major patient safety issue with the hospital environment providing a niche for the rapid evolution of microbial pathogens that are well adapted to infecting susceptible patients. The spore-forming *Clostridium difficile* is one such bacterium, which causes disease in patients undergoing antibiotic therapy. Since 2000, there has been a striking increase in *C. difficile* infections due to the emergence of hypervirulent isolates that appear to possess extended antibiotic resistance and virulence properties. Here we use a genetic approach to identify a factor linked to the development of hypervirulence in *C. difficile*. This study shows that a naturally occurring mutation in a negative regulator of toxin production, the anti-sigma factor TcdC, is an important factor contributing to the development of hypervirulence in epidemic isolates, presumably because it leads to significantly increased toxin production. These results have important implications for *C. difficile* pathogenesis since they suggest that strains carrying a similar mutation have the inherent potential to develop a hypervirulent phenotype. This study has increased our understanding of how these new variant strains cause disease and why they are more harmful, which is critical for the development of improved strategies for preventing and treating these infections.

## Introduction

*C. difficile* is the causative agent of a spectrum of gastrointestinal diseases, collectively known as *C. difficile* infections, or CDI, that are induced by treatment with antibiotics that disrupt the normal gastrointestinal microbiota. CDI can range from mild diarrhoea, through moderately serious disease, to severe life-threatening pseudomembranous colitis, a chronic, often fatal, gastrointestinal disease [1]. During the past decade, there has been an astonishing increase in the rate and prevalence of *C. difficile* infections in many parts of the world, including the UK, USA, Canada and Europe, largely due to the emergence of a “hypervirulent” or epidemic group of isolates belonging to the BI/NAP1/027 category [2], [3]. These strains are highly resistant to fluoroquinolones [3] and are associated with more severe disease and higher mortality rates [4]–[7]. *C. difficile* now also causes disease in those previously not at risk, such as children and pregnant women, with community-associated *C. difficile* disease being increasingly common [8]–[10].

The reasons for the emergence of these strains, and for their increased virulence, remain largely speculative. The use of fluoroquinolones, and the emergence of fluoroquinolone resistant strains, are undoubtedly driving factors in these new epidemics [11], however, the reasons for the heightened virulence and persistence of these strains are unknown. Genotypic and phenotypic comparison of the hypervirulent BI/NAP1/027 isolates to historical strains has identified numerous differences that may contribute to hypervirulence. Phenotypically, these differences may include the production of a toxin known as binary toxin, or CDT [3], and a higher sporulation rate [12]. Whole genome comparisons have identified numerous genetic differences with BI/NAP1/027 strains having an additional 234 genes compared to the well characterised strain 630 [13], including five unique genetic regions that are absent from both strain 630 and non-epidemic 027 strains [4]. Fundamentally, however, the factors directly resulting in the development of hypervirulence by these strains remain unknown.

The major virulence factors of *C. difficile* are two members of the large clostridial cytotoxin family, toxin A and toxin B, encoded by the *tcdA* and *tcdB* genes, respectively, which are potent monoglucosyltransferases that irreversibly modify members of the Rho family of host regulatory proteins [14]. Two recent studies definitively showed that toxin B plays a major role in the virulence of *C. difficile* [15], [16]. The role of toxin A in disease was less clear however, with conflicting data concerning toxin A reported [15], [16].

Epidemic strains are reported to produce significantly more toxin A and toxin B than other strains [2]. The *tcdA* and *tcdB* genes are located on the chromosome within a region known as the pathogenicity locus or PaLoc [17]. In addition to *tcdA* and *tcdB*, the PaLoc encodes three additional genes designated *tcdR*, *tcdE* and *tcdC*, which encode an alternative sigma factor, TcdR [18], a putative holin, TcdE [19], and an anti-sigma factor, TcdC [20], respectively. The expression of toxins A and B is controlled in a complex manner by several factors, including TcdR and TcdC. TcdC is thought to negatively regulate toxin production by interacting with TcdR or with TcdR-containing RNA polymerase holoenzyme or both [20], TcdR is essential for toxin production [18]. BI/NAP1/027 *C. difficile* strains have a nonsense mutation in *tcdC*, which results in the production of a truncated protein that no longer negatively regulates TcdR. This mutation is postulated to be responsible for the increased toxin production observed *in vitro* in these strains [2]. Accordingly, this observation has prompted debate over the importance of the *tcdC* mutation in the hypervirulent phenotype. However, there is currently a lack of experimental evidence to support this hypothesis, with inconsistent reports in the published literature [20]–[22].

Despite their important impact worldwide on public health little is known about the virulence factors of BI/NAP1/027 strains and many important questions about the pathogenesis of disease caused by these strains remain to be answered, especially the role played by TcdC. BI/NAP1/027 isolates have proven difficult to genetically manipulate, which has hampered our ability to study these strains at the molecular level. To address these questions, here we use a novel Tn*916*-based plasmid conjugation system to facilitate the efficient transfer of plasmids into BI/NAP1/027 strains of *C. difficile*. Using this system, we have demonstrated conclusively the role of TcdC as a negative regulator of toxin production in *C. difficile*. Furthermore, using the hamster model of infection, we provide evidence to show that the *tcdC* mutation found in BI/NAP1/027 strains is an important factor in the development of hypervirulence by these strains. This study is the first to use isogenic strains to identify a factor involved in the development of a hypervirulent phenotype in *C. difficile*, and also represents the first *in vivo* demonstration of the role of TcdC in the pathogenesis of *C. difficile* disease.

## Results

### Complementation of the *tcdC* mutation in a BI/NAP1/027 epidemic isolate *in trans*

To determine if mutation of the *tcdC* gene in *C. difficile* BI/NAP1/027 isolates leads to the development of a hypervirulent phenotype it was necessary to construct isogenic BI/NAP1/027 strains that only differed in their ability to produce a functional TcdC protein. To construct the isogenic strains required for this analysis, genetic manipulation of BI/NAP1/027 isolates was required. The genetic manipulation of these strains has proved difficult and attempts to transfer plasmids into BI/NAP1/027 strains using published methods, which rely on RP4-mediated conjugation from *Escherichia coli* [23]–[27], were not successful, even though transfer of plasmids into the genetically amenable strains JIR8094, an erythromycin sensitive derivative of strain 630 [24], and CD37 was readily achieved (Table S1). To overcome this barrier and to facilitate DNA transfer into the strains of interest, we developed a novel plasmid transfer system that exploits the conjugation apparatus encoded by the broad-host range transposon Tn*916*.

The *oriT* region of Tn*916* (*oriT*_Tn*916*_) [28] was cloned into the *catP*-containing *C. difficile* shuttle plasmid pMTL9361Cm [29], generating pDLL4. This plasmid was introduced into *C. perfringens* strain JIR4225, which contains five copies of Tn*916* [30] and plate matings were performed between this donor strain and several *C. difficile* strains, including a BI/NAP1/027 strain, M7404, which is a Canadian epidemic isolate [29]. Transconjugants from these matings were isolated on medium supplemented with thiamphenicol and cefoxitin. The efficiency of plasmid transfer into strain M7404 was 1.2×10^2^–4×10^4^ transconjugants/ml of plated culture. Analysis of transconjugants using PCR specific for the *catP* gene together with restriction analysis confirmed that all putative colonies carried pDLL4 (data not shown), verifying successful plasmid transfer into the BI/NAP1/027 strain M7404. Similar plasmid transfer efficiencies were obtained for numerous other *C. difficile* strains (Table S1), highlighting the utility of this methodology for the genetic manipulation of clinically relevant strains.

To complement the *tcdC* mutation in a BI/NAP1/027 strain the intact *tcdC* gene from strain VPI10463, together with 300 bp of its upstream region, was cloned into the shuttle plasmid pDLL4, generating pDLL17. This plasmid was transferred by Tn*916*-mediated conjugation from *C. perfringens* strain JIR4225 to *C. difficile* strain M7404 as before. PCR was subsequently used to confirm the presence of plasmid pDLL17 in representative transconjugants (data not shown).

**Figure 1:**
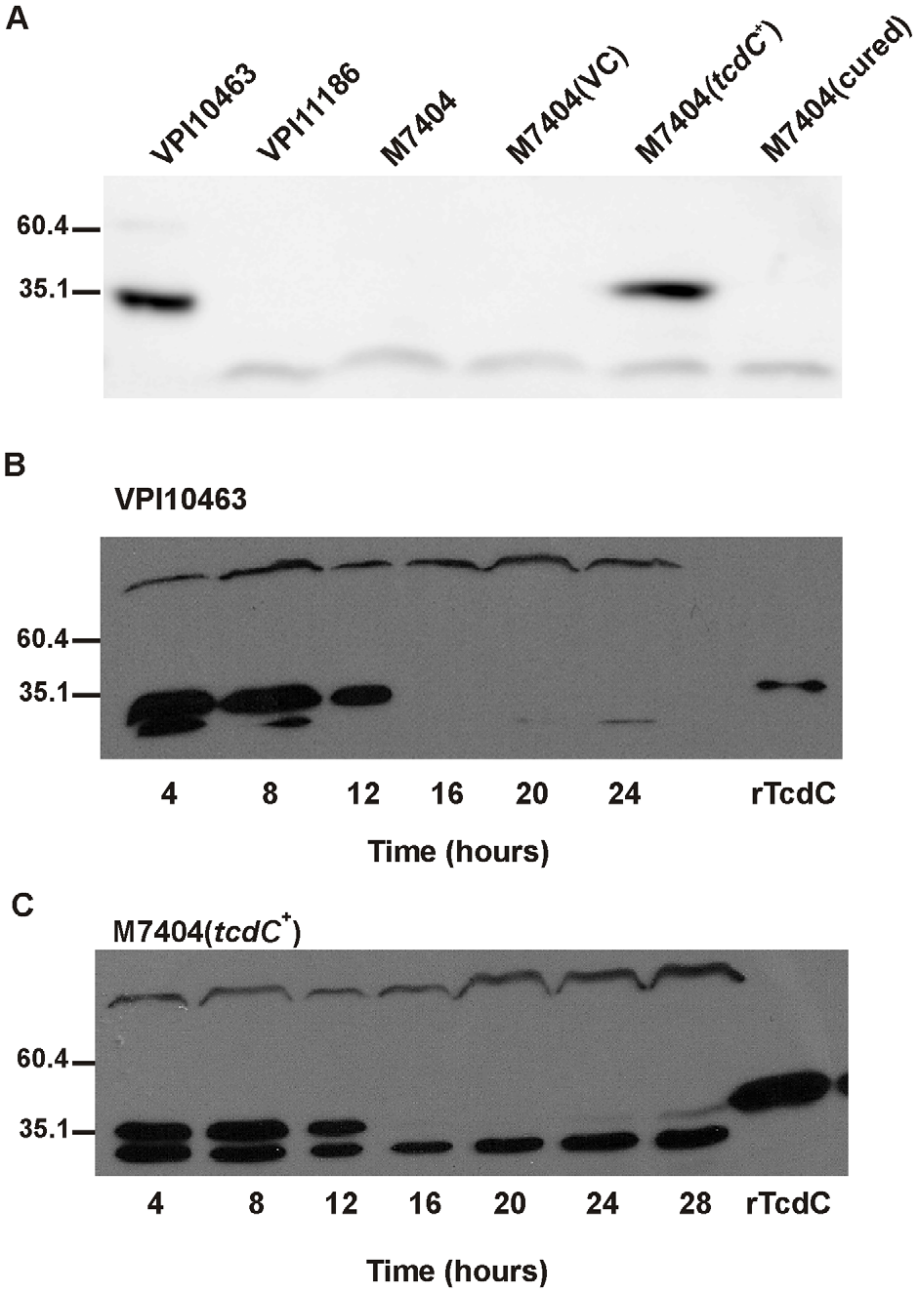
Western blot analysis of TcdC production by wild-type and complemented *C. difficile* strains. (A) Qualitative analysis of TcdC production. VPI10463 is the positive control strain; VPI11186 is the negative control strain; M7404 is a wild-type Canadian BI/NAP1/027 strain; M7404(VC) is the wild-type strain carrying the shuttle plasmid pDLL4; M7404(*tcdC* ^+^) is the wild-type strain carrying the *tcdC* expression plasmid pDLL17 and M7404(cured) is the M7404(*tcdC* ^+^) strain cured of pDLL17. (B) Time course analysis of TcdC production by the positive control strain *C. difficile* VPI10463. Samples were taken at the indicated times shown in hours. 60 ng of purified recombinant his-tagged TcdC protein (rTcdC) was used as the positive reference sample. (C) Time course analysis of TcdC production by *C. difficile* strain M7404(*tcdC* ^+^). Samples were taken at the indicated times shown in hours. 300 ng of purified recombinant his-tagged TcdC protein (rTcdC) was used as the positive reference sample. Western blots were performed with rabbit TcdC-specific antibodies. Size standards are shown (kDa).

To determine whether the presence of pDLL17 complemented the TcdC deficiency of M7404, Western immunoblots using TcdC-specific antibodies were performed. Lysates were collected from the wild-type M7404, the pDLL4-carrying vector control strain M7404(VC) and the pDLL17-*tcdC* ^+^ strain M7404(*tcdC* ^+^), as well as strains VPI10463 and the PaLoc-deficient strain VPI11186, which served as positive and negative controls, respectively. An additional control strain, M7404(cured), was generated by serially passaging strain M7404(*tcdC* ^+^) on non-selective growth medium and curing the plasmid from this strain. Loss of the plasmid was confirmed by sensitivity of the strain to thiamphenicol followed by PCR analysis to verify the absence of several plasmid encoded genes (data not shown). As Figure 1A shows, whilst no TcdC could be detected in the lysates of the negative control strain, the wild-type M7404, M7404(VC) and the plasmid-cured strain M7404(cured), a 34-kDa protein that reacted with TcdC-specific antibodies was detected in lysates from the *tcdC* ^+^-complemented strain M7404(*tcdC* ^+^). This band was the same size as the immunoreactive TcdC protein produced by the positive control strain VPI10463, confirming that the *tcdC* mutation in the BI/NAP1/027 epidemic isolate M7404 was efficiently complemented *in trans*. Since complementation was performed using a multicopy plasmid, we also quantified TcdC production levels from strain M7404(*tcdC* ^+^) in comparison to strain VPI10463 using a time-course assay. Previous studies involving transcriptional analysis of PaLoc genes during different growth phases showed that *tcdC* is expressed in early exponential phase but not in stationary phase, whereas the other PaLoc genes show the opposite expression pattern [31]. VPI10463 (Figure 1B) and M7404(*tcdC* ^+^) (Figure 1C) exhibited similar TcdC expression patterns, with higher levels of TcdC observed in early exponential phase and negligible amounts detected beyond 16 hours, suggesting that the regulatory regions governing *tcdC* expression have been retained on the *tcdC*-carrying fragment used to construct pDLL17. In addition to the kinetics of TcdC expression in strain M7404(*tcdC* ^+^) mirroring that of VPI10463, a similar amount of protein was also detected at each time point with VPI10463 producing 1.3- to 1.6-fold more protein (Figure 1B) than M7404(*tcdC* ^+^) (Figure 1C). Therefore, although *tcdC* complementation was achieved using a multicopy plasmid vector, a physiologically relevant amount of TcdC protein was expressed during the appropriate growth phases in strain M7404(*tcdC* ^+^).

### TcdC-mediated repression of toxin production in *C. difficile*

**Figure 2:**
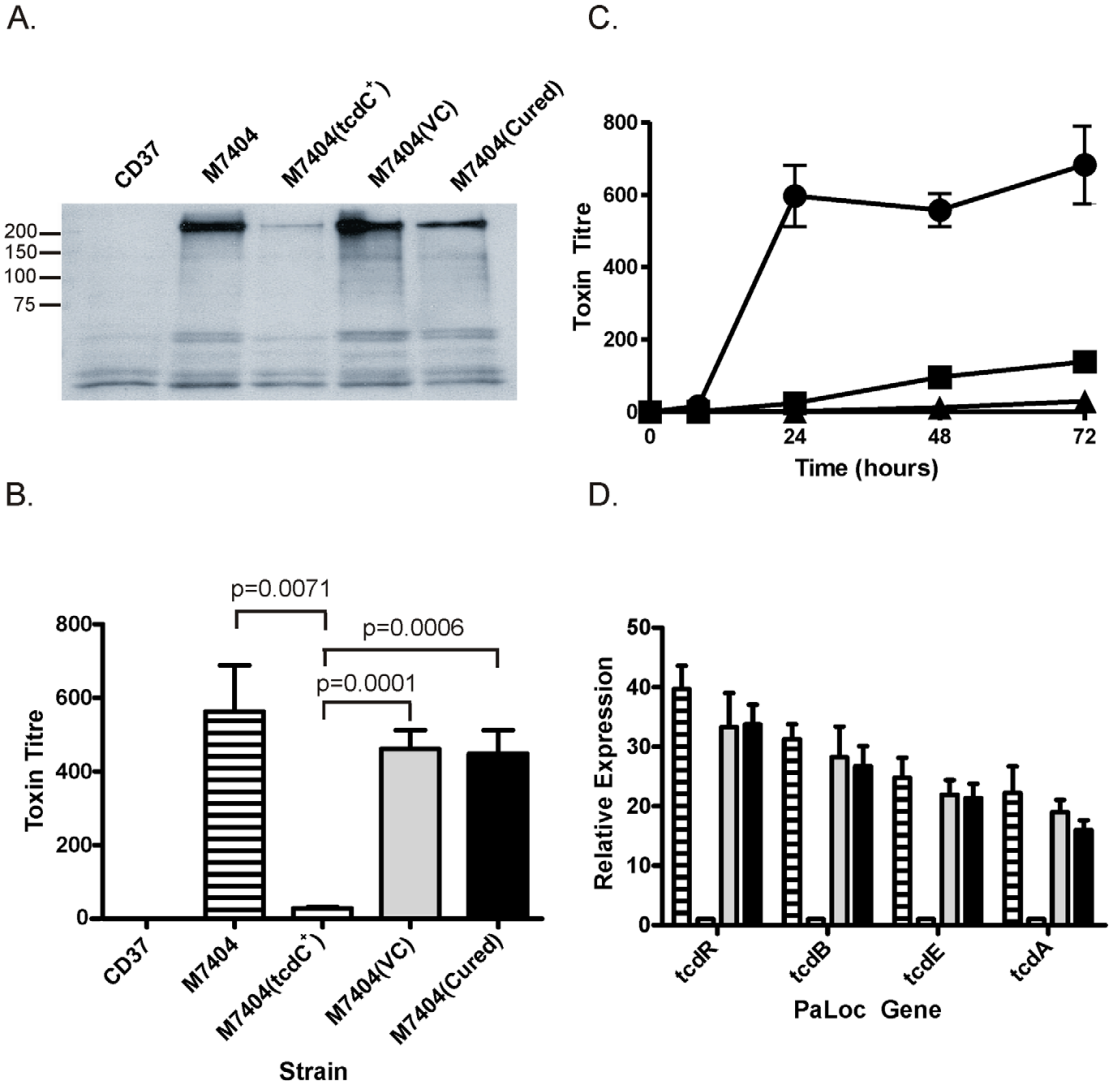
Analysis of the effect of TcdC complementation on toxin production and PaLoc gene expression by *C. difficile*. CD37 is the negative control strain; M7404 is the wild-type BI/NAPI/027 strain; M7404(*tcdC* ^+^) is the wild-type strain carrying the *tcdC* expression vector pDLL17; M7404(VC) is the wild-type strain carrying shuttle plasmid pDLL4 and M7404(cured) is the M7404(*tcdC* ^+^) strain cured of pDLL17. JIR8094 is a derivative of strain 630. (A) Western blot using toxin-A-specific antibodies. Size standards are shown (kDa) (B) Toxin cytotoxicity assays using Vero cells. Strains are as described above. Lined bars represent the wild-type strain M7404; white bars represent M7404(*tcdC* ^+^); grey bars represent M7404(VC) and black bars represent M7404(cured). Data represent the mean ± s.e.m. (n = 3). (C) Time course of toxin production measured using Vero cell cytotoxicity assays. Strains are as described above and are represented as follows: M7404(*tcdC* ^+^) (▪), M7404(VC) (•) and JIR8094 (▴). Note that CD37 was included in this analysis but displayed no toxin production; the line representing this strain is therefore not visible. Data represent the mean ± s.e.m. (n = 3). (D) PaLoc gene specific qRT-PCR. Bars correspond to strains as before and PaLoc genes are indicated. Data represent the mean fold-expression ± s.e.m. (n = 3), compared to the M7404(*tcdC* ^+^) strain.

To determine the effect of TcdC on toxin production in strain M7404, a combination of Western immunoblots and cytotoxicity assays were performed using supernatants collected from strain M7404 and isogenic M7404 derivatives carrying the vector pDLL4, pDLL17, the cured strain M7404(cured) and the PaLoc-negative control strain CD37. To assess toxin A production, Western immunoblotting was performed using TcdA-specific antibodies (Figure 2A). The results showed that the presence of the *tcdC* ^+^ plasmid pDLL17 resulted in a dramatic decrease in the amount of toxin A produced by M7404(*tcdC^+^*) when compared to the wild-type strain. By contrast, M7404(cured) produced qualitatively similar levels of toxin A to wild-type, as did M7404 carrying the vector plasmid, whereas the PaLoc-negative strain CD37 produced no detectable toxin A, as expected.

Vero cell cytotoxicity assays, which predominantly measure toxin B activity [15], were then performed to quantitatively determine the effect of TcdC on toxin production from these strains. As previously observed with toxin A, the amount of toxin produced from strain M7404 was significantly reduced when functional TcdC was restored (Figure 2B). The amount of toxin produced in the TcdC-complemented strain was approximately 16–32-fold less, and therefore significantly lower (p = 0.0001; unpaired t-test, 95% confidence interval), than in the vector control-carrying M7404 derivative. There was, however, no significant difference in toxin activity levels between strains M7404, the vector control strain M7404(VC) or M7404(cured) (Figure 2B). A kinetic analysis of toxin production also clearly showed that the presence of TcdC delayed the onset of toxin production in M7404(*tcdC^+^*) in comparison to M7404 carrying the vector plasmid, mirroring the delayed toxin production observed from the *tcdC* ^+^ 630-strain derivative JIR8094 (Figure 2C).

We also determined if TcdC-mediated repression of toxin production was at the transcriptional level and evaluated the effect of *tcdC* complementation on the expression of the other PaLoc-encoded genes, *tcdR* and *tcdE*. Quantitative real-time PCR (qRT-PCR) analysis using RNA extracted from the wild-type strain and its isogenic derivatives was performed to ascertain the relative transcription levels of the *tcdA*, *tcdB*, *tcdR* and *tcdE* genes. As shown in Figure 2D, an approximate 13- and 23-fold reduction in *tcdA-* and *tcdB-*specific mRNA levels, respectively, in strain M7404(*tcdC* ^+^) was observed compared to M7404. Similar observations were made for *tcdR* and *tcdE* expression levels, with 33-fold and 21-fold less *tcdR* and *tcdE* mRNA, respectively, in the *tcdC*-complemented strain compared to the wild-type. No significant differences in the expression levels of these four genes were detected when M7404, the vector-carrying derivative or the cured strain were compared. These data conclusively demonstrate that TcdC negatively regulates toxin production in *C. difficile* and show that repression occurs at the transcriptional level.

### Reduction of the virulence of a BI/NAP1/027 isolate *via* complementation with TcdC

To define the role of TcdC in the virulence of a BI/NAP1/027 *C. difficile* isolate, female Golden Syrian hamsters were infected with spores of strain M7404 carrying either the vector control or the *tcdC* ^+^ plasmid (n = 10 and n = 12, respectively). For comparative purposes, a group of hamsters (n = 14) was also infected with strain 630, a strain previously characterised as being less virulent than other clinical isolates [32]. Following infection, all *C. difficile* strains were found to be equally efficient at colonising the hamsters (data not shown). Infection of colonised hamsters was allowed to proceed and animals were monitored by telemetry. The end point of infection was achieved when the core body temperature of the hamsters dropped to 35°C. This parameter has previously been shown to be a reliable indicator of non-recoverable disease [33]. At this point, the animals were immediately culled for animal ethics reasons. Bacteria were then isolated from the culled hamsters, the bacterial load quantified and isolates subjected to MVLA analysis [33] to confirm that these isolates were the same strain as originally used for infection.

**Figure 3:**
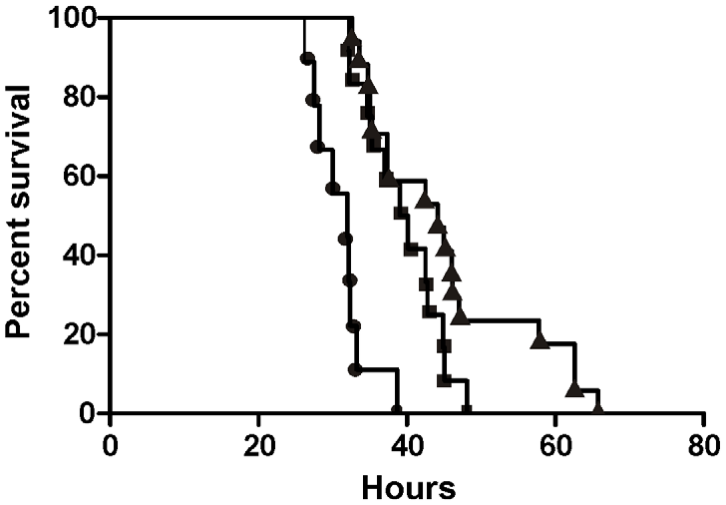
Virulence of *C. difficile* wild-type and *tcdC*-complemented strains in hamsters. Kaplan-Meier survival curve demonstrating time from infection with *C. difficile* to death. M7404(VC), wild-type M7404 carrying shuttle plasmid pDLL4 (•); M7404(*tcdC* ^+^), wild-type M7404 carrying *tcdC* expression plasmid pDLL17 (▪); and strain 630, a *C. difficile* isolate with known low virulence (▴). Hamsters were infected intragastrically with 10,000 spores from each strain; M7404(VC) (n = 9), M7404(*tcdC* ^+^) (n = 12) and strain 630 (n = 14).

Hamsters infected with the M7404(*tcdC* ^+^) derivative showed a significant delay (p = 0.0003; Logrank (Mantel-Cox) test; 95% confidence interval) in the mean time taken to reach non-recoverable disease (2370 minutes or 39.5 hours) in comparison to the vector-carrying M7404 group (M7404(VC)), with a mean time of 1869 minutes or 31.15 hours (Figure 3). In one of the hamsters colonised with the M7404(VC) strain, the time taken to reach the end point of infection was substantially longer than the other hamsters in this group (2814 minutes or 46.9 hours). This hamster was shown by statistical analysis (p = 0.0405; Grubbs test; 95% confidence interval) to be an outlier and was therefore excluded from the experimental analysis. Note that statistical significance would be retained upon inclusion of this outlier. Interestingly, whilst the mean time to the end point of infection in the strain 630 group of hamsters (2701 minutes or 45.02 hours) was significantly longer than that of hamsters infected with M7404(VC) (p = 0.0001; Logrank (Mantel-Cox) test; 95% confidence interval), there was no significant difference in the mean time taken to achieve non-recoverable disease in the 630 group compared to the M7404(*tcdC* ^+^) derivative, indicating that the virulence of the TcdC-complemented strain was equivalent to that of strain 630.

It is apparent from these virulence experiments that the expression of TcdC in a BI/NAP1/027 isolate has an important effect on virulence, resulting in a significant delay in the time needed to reach non-recoverable disease. These data therefore provide compelling evidence that the naturally occurring mutation of *tcdC* in BI/NAP1/027 isolates is an important factor in the development of a hypervirulent phenotype by these strains.

### TcdC status of clinical isolates does not predict toxin production level

The results presented here show that a BI/NAP1/027 strain complemented with *tcdC* is not as virulent as its isogenic vector-carrying control, suggesting that any *C. difficile* strain that acquires a null TcdC phenotype has the potential to develop a hypervirulent phenotype. Furthermore, phylogenetic studies have shown *C. difficile* to be a genetically diverse species, with disease-causing isolates seemingly arising from multiple lineages, suggesting that virulence in these strains may have evolved independently [4], [34]. The *tcdC* status of a diverse group of clinical isolates was therefore determined in parallel to the genetic studies described above. One hundred Australian clinical isolates were initially analysed by toxinotyping, a typing method which categorises strains according to variation in the PaLoc region; BI/NAP1/027 strains belong to toxinotype III [35]. Approximately 5% of these strains were found to belong to a toxinotype associated with a *tcdC* mutation. PCR and sequence analysis was then used to confirm the presence of *tcdC* mutations in each of these isolates. A single BI/NAP1/027 was identified in this survey, designated strain KI [36]. One other strain, DLL3053, was particularly interesting since it belonged to toxinotype group III but was not a BI/NAP1/027 isolate. This strain harboured the well documented single base pair deletion at nucleotide position 117 of *tcdC*, and the 18 base pair in-frame deletion from nucleotide 330 to 347 [37]. Two strains, DLL3054 and DLL3055, were toxinotype V strains with a nonsense mutation at nucleotide position 184 (C184T) and a 39 base pair deletion from nucleotides 341 to 379 [37] whereas strain DLL3056 was toxinotype XIV and possessed a nonsense mutation at nucleotide position 191 (C191A) and an in-frame 36 base pair deletion from nucleotide 300 to 336.

**Figure 4:**
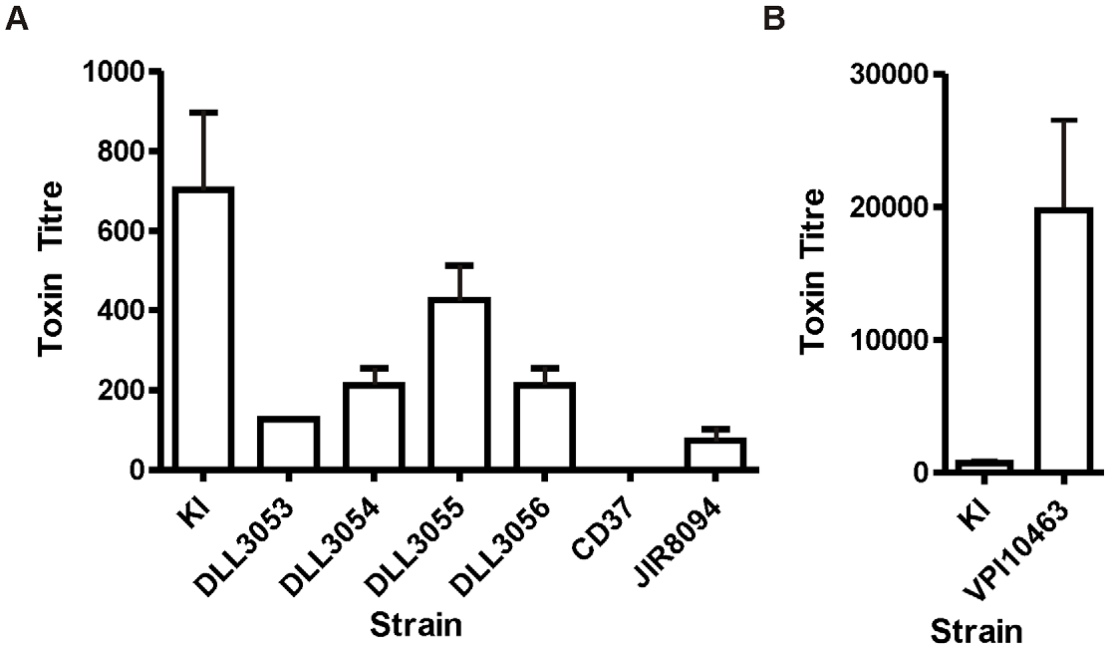
Comparative analysis of toxin production by naturally occurring *tcdC* clinical isolates. Vero cell cytotoxicity assays were used to determine toxin production levels. (A) Strain JIR8094 is a *tcdC* ^+^ control strain, CD37 is a PaLoc-negative control strain and KI is an Australian BI/NAPI/027 isolate [36]. All other strains (DLL3053-DLL3056) are clinical isolates carrying naturally occurring *tcdC* mutations, collected from Australian hospitals. (B) Toxin production by the *tcdC* ^+^ control strain VPI10463 is shown on a separate bar chart due to the much higher levels of toxin produced. For comparative purposes strain KI is represented on both bar charts. Data represent the mean±s.e.m. (n = 3).

To examine the impact of *tcdC* mutations on toxin production in these clinical isolates, Vero cell cytotoxicity assays were performed. These assays, which predominantly detect toxin B, determine the relative amounts of toxin produced by each strain since the expression of toxins A and B is coordinately regulated [23], [31]. Strains JIR8094, a derivative of strain 630 [24], and VPI10463 [38], which both possess intact *tcdC* genes, were used as positive reference controls, and the PaLoc-negative strain CD37 was used as a negative control. Strains were grown in glucose-free medium since glucose has previously been shown to repress toxin production [23]. As shown in Figure 4A, the relative amount of toxin produced by the *tcdC* clinical isolates varied over approximately a 10-fold range. In agreement with the work of others [12], [22], these results show that the presence of mutations within *tcdC* was not directly correlated with high level toxin production. Strain DLL3053 for example, did not produce levels of toxin significantly different from that of strain JIR8094, which is considered to be a low toxin producer [39]. Of all the isolates with *tcdC* mutations, strain KI [36] produced the most toxin, and at significantly (p = 0.0406; unpaired t-test, 95% confidence interval) higher levels that were approximately 10-fold more than strain DLL3053 even though both strains have identical *tcdC* alleles.

In comparison to the TcdC-positive reference strains, all of the *tcdC*-deficient clinical isolates produced more toxin than strain JIR8094 apart from strain DLL3053 (Figure 4A). Conversely, however, all strains produced significantly less toxin than VPI10463 (p = 0.0099–0.0202; unpaired t-test, 95% confidence interval), including the BI/NAP1/027 strain KI. VPI10463 produced over 100-fold more toxin than strains JIR8094 and DLL3053 and over 30-fold more than KI (Figure 4B). This observation is in agreement with recently published findings, which showed that VPI10463 produced significantly more toxin than other strains [12]; however, in that study, strains were grown using glucose-rich BHI medium so differential effects of glucose on toxin production by each strain could not be ruled out. In agreement with other studies [12], [22], [37], our data therefore suggest that the *tcdC*-status alone of *C. difficile* isolates is not an accurate predictor of high-level toxin production.

## Discussion

The hypothesis that the naturally-occurring *tcdC* mutation in epidemic BI/NAP1/027 isolates contributes to hypervirulence is widely accepted, despite a lack of supportive experimental evidence. Indeed, the exact role of TcdC in the pathogenesis of *C. difficile* disease has remained controversial with conflicting findings reported in the literature [20]–[22]. As a result, several published studies have suggested that there is a need to assess isogenic *tcdC* strains in order to conclusively determine the role of this gene in the virulence of *C. difficile* [12], [22], [40]. We have now constructed such isogenic strains and compared them in an animal model. The results conclusively show that TcdC negatively regulates toxin production in *C. difficile*. Most importantly, complementation of the *tcdC* mutation in the BI/NAP1/027 epidemic isolate M7404 clearly showed that this mutation is an important factor in the development of hypervirulence by this strain since the genetic complementation of *tcdC* reduced virulence in comparison to the wild-type strain.

To elucidate the role of TcdC in hypervirulence, it was necessary to construct an isogenic panel of BI/NAP1/027 strains that were identical except for the presence or absence of the wild-type *tcdC* gene. Despite the publication of studies describing the successful transfer of plasmids into the BI/NAP1/027 isolate R20291 [25], [26], [41], this group of strains has remained difficult to work with at the molecular genetic level. As such, a new system that utilised the conjugation apparatus of Tn*916* was developed in this study and used successfully to genetically manipulate a number of clinically relevant isolates, including a BI/NAP1/027 strain of *C. difficile*. Tn*916* is a broad host-range conjugative transposon that was recently used to transfer plasmids into genetically intractable strains of *Enterococcus faecium* [28] and has been shown to transfer into *C. difficile* [42], [43]. *C. perfringens* was chosen for use as a donor strain in anticipation that it may be more proficient for the transfer of plasmids into *C. difficile* in comparison to the more distantly related *E. coli*. The addition of *oriT*_Tn*916*_ onto the shuttle vector pMTL9361Cm facilitated the efficient transfer of this plasmid into strain M7404 from a Tn*916*-carrying *C. perfringens* strain. Furthermore, this system has been successfully used to transfer shuttle plasmids into every *C. difficile* isolate tested so far (Table S1). Most importantly, this new technology facilitated the complementation of the *tcdC* mutation in strain M7404 enabling the role of TcdC in the virulence of BI/NAP1/027 strains of *C. difficile* to be investigated.

Previous *in vitro* studies have shown that TcdC is able to sequester the TcdR sigma factor, preventing its association with core RNA polymerase and blocking toxin gene expression [20]. These experiments suggested that TcdC was important in the regulation of toxin production by *C. difficile*, but the *in vivo* role of this protein was not determined. Conversely, several studies on *C. difficile* clinical isolates [22], [37] showed that the absence of a functional *tcdC* gene was not an accurate predictor of high level toxin production or increased disease severity, indicating that TcdC may not play an important role in virulence in these strains [22], [37]. The analysis of Australian clinical isolates in the present study is in accordance with these latter studies in that isolates with naturally occurring *tcdC* mutations were found to produce toxin at a range of different levels that were not necessarily high. However, since these strains, and those in the other studies [22], [37], are not isogenic it is not possible to draw conclusions about the importance of *tcdC* in the context of toxin yield or virulence. By contrast, the isogenic *tcdC* strains studied here clearly show that TcdC is a negative regulator of toxin production since the *tcdC* complemented BI/NAP1/027 *C. difficile* strain produced significantly less toxin A and B than the non-complemented control strains. The finding that TcdC-status is not correlated with toxin production in clinical isolates highlights the limitation of accurately assigning gene function by studying non-isogenic strains, particularly in a highly heterogeneous species such as *C. difficile*. In this context, it might be of interest to study the function of *tcdC* in isogenic strains generated in a different genetic background such as a ribotype 078 isolate.

Analysis of PaLoc gene expression by qRT-PCR demonstrated that TcdC exerts regulatory control of toxin production at the transcriptional level, and this is in keeping with its proposed role as an anti-sigma factor [20]. The observation that the expression of *tcdR* and *tcdE* is reduced in the *tcdC*-complemented strain, together with *tcdA* and *tcdB*, is probably because of autoregulation of *tcdR* since TcdR upregulates its own expression and that of the other PaLoc genes [23].

The virulence of strain M7404 was reduced upon complementation of *tcdC*, clearly demonstrating that the *tcdC* mutation in BI/NAP1/027 strains has a significant impact on virulence and is likely to be an important factor in the development of hypervirulence by these strains. Surprisingly, the virulence of M7404(*tcdC* ^+^) was found to be equivalent to that of strain 630, which has been shown in other studies to be reduced in virulence in comparison to other isolates, including three other BI-type strains [32]. These findings have important implications for *C. difficile* virulence since they suggest that strains carrying *tcdC* mutations have the inherent potential to develop hypervirulence. The recent emergence of a new class of hypervirulent strains, ribotype 078 [44], may be one such example. These isolates encode a non-functional TcdC protein [37], produce significantly more toxin than non-epidemic strains, are associated with more severe disease as well as higher rates of mortality and are increasingly being identified as the causative agent of CDI [44], [45].

Although these experiments show that TcdC-status alone can modulate virulence it is probable that multiple factors working synergistically are necessary for the development of hypervirulence in the BI/NAP1/027 strains. It is likely that the accumulation of multiple genetic changes in addition to the *tcdC* mutation has enabled BI/NAP1/027 strains to become the predominant disease-causing isolates in numerous countries. Of particular importance might be variations in the functional activity of the encoded toxins since these isolates were recently shown to produce a toxin B that shows variation across the C-terminal receptor binding domain of the protein [46], resulting in more potent activity across a wider range of cell lines in comparison to toxin B from the historical, non-epidemic strain 630 [4]. Furthermore, using the zebrafish embryo model of intoxication, the BI/NAP1/027 toxin B was recently shown to have pronounced *in vivo* cytotoxic activity in comparison to toxin B from VPI10463, another historical non-epidemic isolate, with greater tissue tropism and more extensive tissue destruction observed [47]. Since toxin B is thought to be one of the major virulence factors of *C. difficile* [15], [16] these observations suggest that TcdB variations might play an important role in the hypervirulent phenotype.

There are other factors that may influence *C. difficile* hypervirulence. The BI/NAP1/027 strains encode an additional toxin known as binary toxin or CDT [2]. The role of this toxin in CDI remains to be elucidated but a recent study showed that CDT induces the formation of microtubule-based protrusions on the host cell surface thereby increasing *C. difficile* adherence to epithelial cells. Moreover, intestinal colonisation of gnotobiotic mice with a BI/NAP1/027 *C. difficile* strain was significantly reduced in mice treated with CDT-neutralising antibodies in comparison to control mice [48]. These findings suggest that CDT may be an important colonisation factor, enhancing the ability of BI/NAP1/027 strains to initiate infection as well as causing adjunctive tissue damage during later stages of infection, potentially leading to more severe disease. Many BI/NAP1/027 strains are also more proficient at sporulation than non-epidemic *C. difficile* strains [12], [49]. *C. difficile* spores are highly infectious [50] and play a critical role in the transmission of CDI and perhaps in disease relapse, which is a serious problem in patients with CDI [51]. In this context, enhanced sporulation is ostensibly an important adaptation by BI/NAP1/027 isolates, which would result in larger numbers of spores being shed from infected patients and an increased environmental spore load, ultimately leading to higher transmission rates. Finally, the development of fluoroquinolone resistance, in particular to moxifloxacin and gatifloxacin, is unquestionably a major factor in epidemics caused by BI/NAP1/027 strains [3], [11]. In this regard, the hypothetical co-evolution of enhanced virulence traits and antibiotic resistance in *C. difficile* mirrors trends seen with other significant nosocomial pathogens such as Methicillin Resistant *Staphylococcus aureus* (MRSA) [52] and Vancomycin Resistant *Enterococcus* (VRE) species [53]. In summary, it is clear that our findings represent an important breakthrough in our understanding of the development of hypervirulence in prevailing *C. difficile* isolates and will provide a significant reference point for future studies on epidemic strains and their control.

## Materials and Methods

### Ethics statement

This study was carried out in strict accordance with the recommendations in the United Kingdoms Home Office Animals (Scientific Procedures) Act of 1986 which outlines the regulation of the use of laboratory animals for the use of animals in scientific procedures. The experiments described were subject to approval by the University of Glasgow Ethics Committee and by a designated Home Office Inspector (Project Number 60/4218). All experiments were subject to the 3 R consideration (refine, reduce and replace) and all efforts were made to minimize suffering.

### Bacterial strains and growth conditions

The characteristics and origins of all recombinant strains and plasmids are shown in Table 1 and Table S2, respectively. All bacteriological culture media were obtained from Oxoid. *C. difficile* strains were cultured in BHIS [54] or TY medium [15], unless otherwise stated, in an atmosphere of 10% H_2_, 10% CO_2_, and 80% N_2_ at 37°C in a Coy anaerobic chamber. *Escherichia coli* was cultured in 2×YT medium aerobically at 37°C, with shaking for broth cultures. All antibiotics were purchased from Sigma-Aldrich and were used at the following concentrations: cycloserine (Cs, 250 µg/ml), cefoxitin (Cf, 8 µg/ml), thiamphenicol (Tm, 10 µg/ml) or tetracycline (Tc, 10 µg/ml), chloramphenicol (Cm, 25 µg/ml).

**Table 1:** Bacterial strains.

Strain	Characteristics	Source/Reference
***E. coli***		
DH5α	F-φ80Δ *lacZ*dM15Δ(*lacZYA*-*argF*)U169 *endA1recA1 hsdr17*(τ_κ_^−^m_κ_^+^)*deoR thi-1 supE44 gyrA96 relA1*	Life Technologies
TOP10	F- *mcr*A Δ(*mrr-hsd*RMS-*mcr*BC) φ80*lac*ZΔM15 Δ*lac*X74 *nup*G *rec*A1 *ara*D139 Δ(*ara-leu*)7697 *gal*U *gal*K *rps*L(Str^R^) *end*A1 λ^−^	
	Invitrogen	
HB101	*thi*-*1 hsdS20*(r_B_^−^ m_B_^−^) *supE44 recAB ara-14 leuB5 proA2 lacY1 galK rpsL20* (Sm^r^) *xyl-5 mtl-1*	[60]
***C. perfringens***		
JIR4225	*C. perfringens* strain JIR325 with 5 chromosomal copies of Tn*916*	[30]
***C. difficile***		
M7404	Canadian BI/NAP1/027 isolate	[29]
630	Wild-type *C. difficile* strain; first *C. difficile* genome available	[61]
JIR8094	Erythromycin sensitive derivative of strain 630	[24]
VPI10463	PaLoc-positive *C. difficile* isolate	[38]
VPI11186	PaLoc-negative *C. difficile* isolate	[38]
CD37	PaLoc-negative *C. difficile* isolate	[62]
KI	Australian BI/NAP1/027 isolate	[36]
DLL3053	Australian toxinotype III clinical isolate	This study
DLL3054	Australian toxinotype V clinical isolate	This study
DLL3055	Australian toxinotype V clinical isolate	This study
DLL3056	Australian toxinotype XIV clinical isolate	This study
M7404(VC)	DLL3001 (M7404 carrying shuttle plasmid pDLL4)	This study
M7404(*tcdC* ^+^)	DLL3002 (M7404 carrying *tcdC* expression plasmid pDLL17)	This study
M7404(cured)	DLL3003 (M7404(*tcdC* ^+^) cured of plasmid pDLL17)	This study

### Molecular biology and PCR techniques

Plasmid DNA was isolated using a QIAprep spin miniprep kit (Qiagen). Genomic DNA was prepared using a DNeasy tissue kit (Qiagen). Standard methods for the digestion, modification, ligation, and analysis of plasmid and genomic DNA were used [55]. Nucleotide sequence analysis was carried out using a PRISM BigDye Terminator cycle sequencing kit (Applied Biosystems) and detection was performed by Micromon at Monash University. Oligonucleotide primer sequences are listed below. Unless otherwise stated, all PCR experiments were carried out with Phusion DNA polymerase (New England Biolabs) and the 2× Failsafe PCR buffer E (Epicentre) according to the manufacturer's instructions.

### Construction of recombinant plasmids

For construction of the Tn*916* transferrable clostridial shuttle vector, PCR was performed using primers DLP33 (5′-GAATTCGCCCTTTTTTATACTCCCCTTG-3′) and DLP34 (5′-GAATTCGCCCTCAAAGGACGAATATGTCGC-3′) and chromosomal DNA extracted from *Clostridium perfringens* strain JIR4225 [30]. The resulting 700 bp DNA fragment, which contained the *oriT* region of Tn*916*, was TOPO-cloned into pCR-Blunt II-TOPO according to the manufacturer's instructions (Invitrogen). The fragment was then excised from pCR-Blunt II-TOPO using *Eco*RI and cloned into the equivalent sites of plasmid pMTL9361Cm [29], resulting in plasmid pDLL4.

For construction of the *tcdC*-carrying plasmid, PCR was performed using primers DLP35 (5′-CTGCAGCCACCTCTAAATCACTGAGTCACTTAATTAC-3′) and DLP36 (5′-CTGCAGAGCCTTGTAACTGTTTATTTGC-3′) and *C. difficile* strain VPI10463 genomic DNA in order to amplify a 1085 bp fragment encompassing the *tcdC* gene and upstream region. This fragment was then TOPO-cloned into pCR-Blunt II-TOPO, before being excised with *Pst*I and subcloned into the equivalent site of plasmid pDLL4, resulting in the final construct pDLL17.

### Transfer of plasmid DNA into *C. difficile* by conjugation

The conjugation procedure utilising *E. coli* HB101(pVS520) as the conjugative donor was carried out as previously described [29]. Recombinant plasmids were introduced into *C. perfringens* strain JIR4225 as before [56]. Conjugations utilising *C. perfringens* JIR4225 were then performed as follows: separate 90 ml BHIS broth cultures were inoculated with 1 ml aliquots from an overnight *C. difficile* recipient strain or *C. perfringens* donor strain starter culture and grown to mid-exponential phase. Approximately 1 ml was removed from each culture, mixed and centrifuged. The cell pellet was then resuspended in phosphate-buffered saline (PBS), spread onto a BHIS agar plate and incubated overnight at 37°C. Bacterial growth was harvested in sterile PBS before being spread onto BHIS agar supplemented with thiamphenicol and incubated overnight as before. Bacterial growth was again harvested with PBS and dilutions spread onto BHIS agar supplemented with cefoxitin and thiamphenicol or tetracycline, and the plates incubated under anaerobic conditions for 24 to 72 h.

### Toxin A-specific Western blots

The toxins were partially purified by ammonium sulphate precipitation from culture supernatants harvested after growth for 72 hours and toxin A was then detected by Western blotting as described previously [15].

### TcdC-specific Western blots

For non-quantitative TcdC-specific Western Blots, crude extracts of *C. difficile* were prepared by sonication of samples taken from cultures that had been grown for 12 hours under anaerobic conditions. The crude extracts from each strain were then subjected to electrophoresis in a 15% SDS-PAGE gel and transferred to a nitrocellulose membrane using standard methods [55]. Membranes were treated with anti-TcdC antibody [57] and detected following treatment with goat anti-mouse IgG-alkaline phosphatase conjugated secondary antibody using standard procedures. For quantitative TcdC-specific Western blots, cultures of *C. difficile* VPI10463 and *C. difficile* M7404(*tcdC* ^+^) were grown under anaerobic conditions and samples were removed every 4 hours for 24 or 28 hours respectively. Each sample was normalized to an optical density (600 nm) of 0.9 prior to lysis, to ensure that the same number of cells was present. Lysates were then prepared by sonication prior to SDS-PAGE gel electrophoresis, transfer and detection, as described above. Following detection, the amount of TcdC in each lysate was quantified by densitometric analysis, using purified recombinant TcdC protein (rTcdC) as the standard and the ImageJ software package, according to published methods [58].

### Vero cell cytotoxicity assays

Toxin B was detected in *C. difficile* culture supernatants harvested after growth for 72 hours by Vero cell cytotoxicity assays as described previously [15], except that each well was seeded with 1×10^5^ cells.

### RNA extraction and reverse transcription

Total RNA was extracted from *C. difficile* cultures grown for 12 h in TY media. Reverse transcription was performed using AMV Reverse Transcriptase (Promega) using random hexamer oligonucleotides primers and 2 µg template RNA. The cDNA samples were then purified using Qiaquick Columns (Qiagen).

### qRT-PCR assay design and real time PCR

PaLoc gene specific primers were designed using Primer 3 software (Geneious Software). qRT-PCR was performed using an AB7300 real-time PCR instrument (Applied Biosystems). Reactions were carried out using the FastStart Universal SYBR Green Master Mix (Roche) with 40 ng of cDNA as template. Standard curves were generated for each primer pair using *C. difficile* genomic DNA, and melt curve analysis was performed following each qRT-PCR reaction to verify amplification specificity. Samples were normalised using the *C. difficile rrnA* gene.

### Preparation of spores for animal infection

Spores were prepared from *C. difficile* cultures grown in 500 ml of BHI broth. Cultures were pelleted by centrifugation for 10 mins and re-suspended in 50% ethanol. The material was then vortexed every 10 min for 1 h before centrifugation for 10 mins. The pellet was then treated with 1% Sarkosyl in PBS for 1 h at room temperature and again pelleted by centrifugation, followed by incubation overnight at 37°C with lysozyme (10 mg/ml) in 125 mM Tris-HCl buffer (pH 8.0). The sample was treated in a sonicating water bath (3 pulses of 3 min each; 1510 Branson) before centrifugation through a 50% sucrose gradient for 20 mins. The pellet was incubated in 2 ml of PBS containing 200 mM EDTA, 300 ng/ml proteinase K and 1% Sarkosyl for 30 mins at 37°C before centrifugation through a 50% sucrose gradient for 20 mins. The final pellet was then washed twice in sterile distilled water before finally being resuspended in 1 ml of sterile water. Spore preparations were stored at −80°C prior to use.

### Hamster experiments

Female Golden Syrian hamsters purchased from Harlan Olac UK were used for all animal experiments. Telemetry chips (Vitalview Emitter) were inserted by laparotomy into the body cavity of the animals at least 3 weeks before infection with *C. difficile*. Animal experiments were then carried out as described previously [33], except that animals received 1×10^4^ spores of *C. difficile*. Animals were culled when core body temperature dropped below 35°C. This study was carried out in strict accordance with the recommendations in the United Kingdoms Home Office Animals (Scientific Procedures) Act of 1986 which outlines the regulation of the use of laboratory animals for the use of animals in scientific procedures. The experiments described were subject to approval by the University of Glasgow Ethics Committee and by a designated Home Office Inspector (Project Number 60/4218). All experiments were subject to the 3 R consideration (refine, reduce and replace) and all efforts were made to minimize suffering.

### Quantification of bacterial load

To estimate colonisation, hamsters were sacrificed and the gut region from the caecum to the anus removed. The tissues were homogenised in PBS using a Stomacher and viable counts were performed on the homogenate as described previously [33].

### Confirmation of infecting strains

To confirm that the bacteria isolated from the hamster were the same strain as originally used for infection, genomic DNA was isolated and subjected to MVLA as described previously [59]. Plasmid rescue was performed as previously described [29] followed by restriction digest analysis to confirm plasmid integrity.

## Supporting Information

Table S1 **Efficiency of RP4 or Tn** ***916*** **-mediated plasmid transfer to** ***C. difficile*** **strains.** The efficiency of RP4 or Tn*916*-mediated plasmid transfer from *E. coli* or *C. perfringens* donors, respectively, to *C. difficile* recipient strains is shown, calculated as described in Materials and Methods and expressed as transconjugants per ml.
(DOC)

Table S2 **Bacterial plasmids.** Bacterial plasmids used in this study, together with the genetic features relevant to this work, are shown.
(DOC)
