# Supplementary material for: The Anti-Sigma Factor TcdC Modulates Hypervirulence in an Epidemic BI/NAP1/027 Clinical Isolate of Clostridium difficile
Source: PLoS Pathog. 2011 Oct 13;7(10):e1002317. doi: 10.1371/journal.ppat.1002317 (PMC3192846; doi:10.1371/journal.ppat.1002317)
Supplement: Table S1 — Efficiency of RP4 or Tn 916 -mediated plasmid transfer to C. difficile strains. The efficiency of RP4 or Tn916-mediated plasmid transfer from E. coli or C. perfringens donors, respectively, to C. difficile recipient strains is shown, calculated as described in Materials and Methods and expressed as transconjugants per ml. (DOC) [file ppat.1002317.s001.doc]

| **Strain** | **Toxinotype** | **Efficiency of RP4-mediated transfer (transconjugants/ml)** | **Efficiency of Tn*916*-mediated transfer (transconjugants/ml)** | **Source** |
| --- | --- | --- | --- | --- |
| JIR8094 | 0 | 2.3 x 10^2^ – 3 x10^3^ | 1.2 x 10^1^ – 4 x 10^3^ | [1] |
| CD37 | non-toxigenic | 4 x 10^1^ – 2.4 x 10^3^ | 2 x 10^2^ – 3 x 10^3^ | [2] |
| VPI10463 | 0 | 0 | 1.6 x 10^3^- 2.9 x 10^3^ | [3] |
| M7404 | III | 0 | 4 x 10^1^ – 2.5 x 10^3^ | [4] |
| R20291 | III | 0 | 2 x 10^1^ – 1.7 x 10^3^ | [5] |
| R20352 | III | 0 | 1.2 x 10^1^- 6.7 x 10^2^ | [5] |
| R12087 | III | 0 | 3.6 x 10^2^ – 1.8 x 10^3^ | [5] |
| CD196 | III | 0 | 8.1 x 10^2^- 3.8 x 10^3^ | [6] |
| JGS6133 | V | 0 | 7.1 x 10^2^- 2.6 x 10^3^ | J. G. Songer; Iowa State University. |

**Table S1. Efficiency of RP4 or Tn*916*-mediated plasmid transfer to *C*. *difficile* strains.**

1. O'Connor JR, Lyras D, Farrow KA, Adams V, Powell DR, et al. (2006) Construction and analysis of chromosomal *Clostridium difficile* mutants. Mol Microbiol 61: 1335-1351.

2. Mullany P, Wilks M, Lamb I, Clayton C, Wren B, et al. (1990) Genetic analysis of a tetracycline resistance element from *Clostridium difficile* and its conjugal transfer to and from *Bacillus subtilis*. Journal of General Microbiology 136: 1343-1349.

3. Lyerly DM, Barroso LA, Wilkins TD, Depitre C, Corthier G (1992) Characterization of a toxin A-negative, toxin B-positive strain of *Clostridium difficile*. Infect Immun 60: 4633-4639.

4. Carter GP, Lyras D, Allen DL, Mackin KE, Howarth PM, et al. (2007) Binary toxin production in *Clostridium difficile* is regulated by CdtR, a LytTR family response regulator. Journal of Bacteriology 189: 7290-7301.

5. Stabler RA, Gerding DN, Songer JG, Drudy D, Brazier JS, et al. (2006) Comparative phylogenomics of *Clostridium difficile* reveals clade specificity and microevolution of hypervirulent strains. Journal of Bacteriology 188: 7297-7305.

6. Perelle S, Gibert M, Bourlioux P, Corthier G, Popoff M (1997) Production of a complete binary toxin (actin-specific ADP-ribosyltransferase) by *Clostridium difficile* CD196. Infection and Immunity 65: 1402-1407.
