# Supplementary material for: The Anti-Sigma Factor TcdC Modulates Hypervirulence in an Epidemic BI/NAP1/027 Clinical Isolate of Clostridium difficile
Source: PLoS Pathog. 2011 Oct 13;7(10):e1002317. doi: 10.1371/journal.ppat.1002317 (PMC3192846; doi:10.1371/journal.ppat.1002317)
Supplement: Table S2 — Bacterial plasmids. Bacterial plasmids used in this study, together with the genetic features relevant to this work, are shown. (DOC) [file ppat.1002317.s002.doc]

**Table S2. Bacterial plasmids.**

| **Plasmid** | **Characteristics** | **Source/Reference** |
| --- | --- | --- |
|  |  |  |
| pVS520 | Tra^+^ Mob^+^ RP4 derivative, Tc^r^ | [1] |
| pMTL9361Cm | *E. coli-C. difficile* mobilizable  shuttle vector, carries pCD6  replication region, *oriT_RP4_* and  Tm^R^ | [2] |
| pDLL4 | pMTL9361Cm (*Eco*RI) DLP33/DLP34 PCR product (*Eco*RI, 700 bp) (*oriT*_Tn_*_916_* inserted into *E. coli-C. difficile* shuttle vector allows plasmid to be mobilised by Tn*916*), Tm^R^ | This study |
| pDLL17 | pDLL4 (*Pst*I) DLP35/ DLP36 PCR product (*Pst*I; 1085 bp) (*C. difficile tcdC* expression vector), Tm^R^ | This study |
|  |  |  |

1. Palombo EA, Yusoff K, Stanisich VA, Krishnapillai V, Willetts NS (1989) Cloning and genetic analysis of *tra* cistrons of the Tra 2/Tra 3 region of plasmid RP1. Plasmid 22: 59-69.

2. Carter GP, Lyras D, Allen DL, Mackin KE, Howarth PM, et al. (2007) Binary toxin production in *Clostridium difficile* is regulated by CdtR, a LytTR family response regulator. Journal of Bacteriology 189: 7290-7301.
